# Changes in global Orchidaceae disease geographical research trends: recent incidences, distributions, treatment, and challenges

**DOI:** 10.1080/21655979.2020.1853447

**Published:** 2020-12-21

**Authors:** Archana Jain, Surendra Sarsaiya, Jishuang Chen, Qin Wu, Yuanfu Lu, Jingshan Shi

**Affiliations:** a Key Laboratory of Basic Pharmacology and Joint International Research Laboratory of Ethnomedicine of Ministry of Education, Zunyi Medical University, Zunyi, Guizhou, China; b Bioresource Institute for Healthy Utilization, Zunyi Medical University, Zunyi, Guizhou, China; c Laboratory, CES Analytical and Research Services India Private Limited (Formerly Known as Creative Enviro Services), Bhopal, Madhya Pradesh, India

**Keywords:** Orchidaceae, geographical distribution, diseases, detection, environmental conditions, pathogens

## Abstract

Many of the Orchidaceae species are threatened due to environmental changes and over exploitation for full fill global demands. The main objective of this article was critically analyzed the recent global distribution of Orchidaceae diversity, its disease patterns, microbial disease identification, detection, along with prevention and challenges. Critical analysis findings revealed that Orchidaceae growth and developments were affected indirectly or directly as a result of complex microbial ecological interactions. Studies have identified many species associated with orchids, some are pathogenic and cause symptoms such as soft rot, brown rot, brown spot, black rot, wilt, foliar, root rot, anthracnose, leaf spot. The review was provided the comprehensive data to evaluate the identification and detection of microbial disease, which is the most important challenge for sustainable cultivation of Orchidaceae diversity. Furthermore, this article is the foremost of disease triggering microbes, orchid relations, and assimilates various consequences that both promoted the considerate and facts of such disease multipart, and will permit the development of best operative disease management practices.

## GRAPHICAL ABSTRACT

**Figure:**
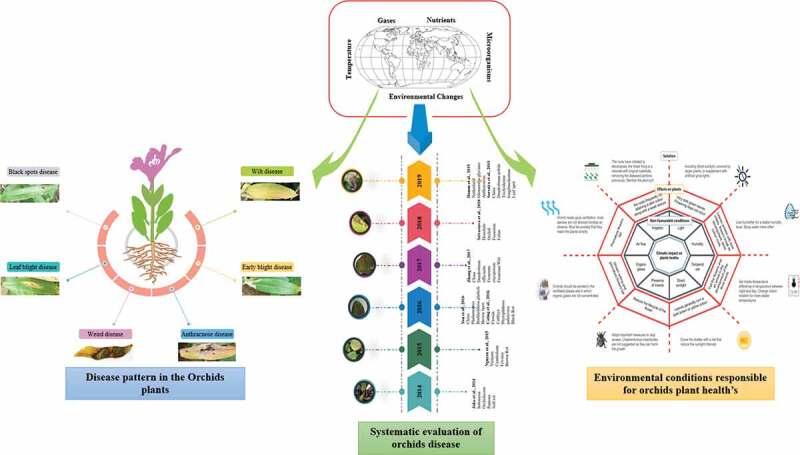

## Introduction

Orchidaceae is the most significant Chinese therapeutic family of plants, with more than 28,000 recognized plants in around 763 genera. The chief Orchidaceae genera are *Pleurothallis* (species: over 1000), *Dendrobium* (species: over 1400), *Epidendrum* (species: over 1500), and *Bulbophyllum* (species: over 2000) [1,2]. Some Orchidaceae species are inattentive due to Arctic parts and desert regions but are chiefly abundant in the wet humid regions of wide-reaching [3,4]. Orchidaceae is one of the most dissimilar groups of blooming plants, happening on totally vegetated landmasses and even some Antarctic islands. The Orchidaceae family has a high percentage of susceptible genera, with most holding threatened species. Orchidaceae is occurred in nature habitat, mainly spread in east Asia, South-East Asia and Oceania [5].

Orchids are recognized as a economic crops significantly. With expanding around the world request for these excellent and intriguing plants, numerous nations have started developing and trading orchids. Expanded generation and worldwide exchange have driven to the development of plants. Information of degree and structure of species hereditary differing quality is fundamental for the foundation of an effective preservation procedure since hereditary variables contribute to species termination chance through inbreeding depression, misfortune of hereditary differences, and loss of developmental potential. On the other hand, high population hereditary variety is another critical basis for prioritization of populaces for protection. Next, to its commitment to the entire species hereditary differences, adequately high within-population hereditary variety is significant for a species populace long-term survival and capability to reply and adjust to the natural changing situation’s changes [6].

The most common diseases of orchid plants are caused by fungal species. These might be foliar blights, leaf spots, fungal rots, and flower blights. The bacterial rot can diminish orchid health. The determination of plant disease is found critical to treat orchid diseases [7]. Most orchid infections can be avoided or cured particularly if caught early. Just as with bugs, it is critical to screen plant health regularly and acts quickly on the chances that any unusual conditions happen. A few orchids could be generalists, tolerating mycorrhizae representing a few diverse species whereas others could be specialists, undertaking connections with only one or a pair of related species. The simplification or specificity of these advantageous associations may or may not dictate dissemination and plenitude of orchids within the wild. Orchidaceae is amplified green plants via two improvement courses of action: sympodial improvement, where a modern shoot advancement as of profound rooted shoot part; or monopodial improvement. *Fusarium* species are accountable for foliar in addition to root sicknesses on Orchidaceae due to *F. fractiflexum, F. proliferatum* and *F. subglutinans* [3].

In some conditions, the Orchidaceae diseases are caused via plant-pathogenic fungi. These are leaf spot (Causal agent: *Nigrospora oryzae*), leaf spot (Causal agent: *Cladosporium cladosporioides*), wilt (Causal agent: *Fusarium oxysporum*), blight with root rot (Causal agent: *Phytophthora capsica*), anthracnose (Causal agent: *Colletotrichum gloeosporioides*), black spot (Causal agent: *Alternaria alternata*), leaf spots (Causal agent: *Phyllosticta capitalensis*), and leaf spots (Causal agent: *Phoma multirostrata*). It is exceptionally mutual to peach many strain taking place in the comparable region, triggering corresponding disease symptoms. Although, the changeover from pathogens to non-pathogenic microbes and vice versa could not primarily be a long movement but can emerge by transformation or erasure of a mono gene [8]. Indeed, more quickly, the endophytes can be changed over pathogenic by just altering the development situations of the Orchidaceae plants, which are too identified as ‘conditional pathogens [3]. The existing method for orchid’s diseases are based on the proof of identity and recognition of pathogens by evaluation with existing databases (national, international or regional information systems). Though, such methods are not fully reliable. Nanotechnology has got tremendous implications in rapid detection of plant pathogens, biosensor-related control of pest and diseases, soil management, etc [9]. Details of current research trends, including the incidences and geographical distribution are critically covered in this article. The review has been given the recent global distribution of Orchidaceae diversity along with its disease patterns, microbial disease identification, and detection, along with prevention and challenges.

## Geographical distribution of orchid diversity

The Orchidaceae is the chief blossoming plant’s family, counting more than 763 genera beside more than 28,000 species [2]. They, moreover, represent the second-largest blossoming plant family in India, with 1,141 species in 166 genera, and contribute generally 10% of Indian greenery. Guo et al. [6] have depicted the population genetic structure in an undermined orchid *Cypripedium tibeticum* from the foremost broad species of *Cypripedium* and is native to the East Himalaya Hengduan Mountains (China). It is found that *Cypripedium tibeticum* could be a debilitated orchid which productive preservation requires information of its degree and structure of a hereditary variety. *C. tibeticum* had been high added up to be hereditary differing from qualities with major commitment to this differing quality made by among-population component. In other cases, in spite of high populace separation, there was no clear phylogeographic structure. The populace *Cypripedium* had the most noteworthy level of nucleotide differing qualities as well as allelic abundance. Subsequently, these two populaces ought to have the best need in preservation arranging and usage. This species is ordinarily clustered in inadequate ecosystem at high elevations [6].

Chen et al. [5] have depicted the hereditary differences and populace structure of the therapeutic Orchid *Gastrodia elata* from eastern Sichuan to western Hubei. It is found that the wild assets of *Gastrodia elataare* as of now undermined with termination due to over harvesting since of their high therapeutic esteem. Hereditary differences play a main part within the survival of imperiled orchid species. The populaces with a tall level of hereditary differences or with awesome hereditary qualification were distinguished, which ought to be a tall need for preservation supervisors. Clear hereditary structure was found among the *Gastrodia elata* populaces, and the extent of hereditary separation among populaces accounted for around 20% of add up to be hereditary differing from qualities [10]. The wild *Gastrodia elata* populaces appeared a generally low level of hereditary differing qualities and self-evident hereditary structure. All the populaces should be secured, particularly the populaces with a tall level of hereditary differing qualities or with extraordinary hereditary refinement. Hereditary variety is critical for a species to preserve its developmental potential to manage with ever-changing situations. Also, as a well-known herb in East Asia, wild assets of *Gastrodia elata* are critical supplies of qualities for moving forward commercial assortments. The data picked up around the levels and conveyance of smaller-scale satellite variety within the therapeutic orchid; *Gastrodia elata* can be utilized to propose suitable management procedures [5].

Chinsamy et al. [11] have portrayed the antioxidant, anti-inflammatory, anti-cholinesterase action and mutagen city of South Africa restorative orchids. It is found that the part of different forms in inflammatory-related degenerative clutters is still being investigated; numerous roads of research have concentrated on the treatment and/or anticipation of these clutters. Inflammatory-responses, the cholinergic framework, and oxidative push have regularly been connected to the side effects predominant in matured people. South African conventional medication was moreover, included as they were being exchanged within the above-mentioned herbal markets. Most of the orchid species utilized for social practices are managed as emetics. It would be imperative to know what the impacts of these orchid-derived drugs are on the human body and more particularly, their security. The employments of certain orchid species are conventional in pharmaceutical for pain-related sicknesses in South Africa. A more comprehensive appraisal of the chemistry of South African orchids would permit one to more certainly state a relationship between chemical profiles, interaction between diverse classes of compounds, organic action and impact of the geological area [11].

Nguyen et al. [12] have depicted the four Tulasnella taxa related to populaces of the Australian evergreen earthbound orchid *Cryptostylis ovata*. The hereditary difference inside the southern populace was less than that of the northern gather, where three potential species happened. It is found that ITS (internal transcribed spacer) locales of Tulasnella separate collected from a few genera of Australian earthly orchids, but not counting *Cryptostylis*, were biased between species. *Cryptostylis ovatais* is the alone plant species that holds its leaves throughout the year circular. It exists as an earthly herb and once in a while as an epiphyte in forest regions. One *Tulasnella* cluster was a display only within the three orchid southern populaces, and it has strictly taken after *T. prima* segregates already depicted from *Chiloglottis* species orchids from the east part of Australia. The isolates collected from plants within the two northern populaces were undescribed *Tulasnella bunches* [13].

**Figure 1.**
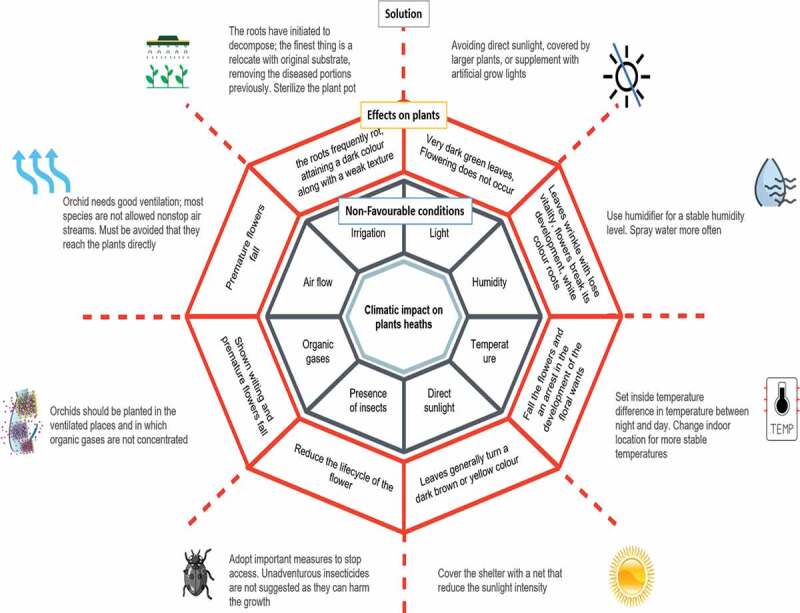
Systematic overview of environmental conditions responsible for orchids plant health’s

Alzate-Q et al. [14] have depicted the impact of land utilize sorts on the composition and differing qualities of orchids and their phorophytes in forest parts from central Veracruz, Mexico. It is found to characterize the differing qualities of earthly and epiphytic orchids, as well as their phorophytes, in six destinations found in three territories having distinctive sorts of arrive utilize: mature forest (MF), riparian forest (RF) belts crossing open pastures, and shaded coffee (SC) manors. The sort of arrive to utilize, especially the nearness of woodland parts or huge trees inside rural scenes beneath, the differences of orchids are diminished with expanding human-centered unsettling influences. It is anticipated within the destinations with more noteworthy unsettling influence. There will be a more prominent impact on the environmental conditions and thus on the composition, abundance, and wealth of orchids as well as on their vertical and even dissemination [14].

Martins et al. [15] have described the tree from tops to the ground: inversions to earthbound propensity in Galeandra orchids (*Epidendroideae: Catasetinae*) from Brazil. It is found in the root for the enhancement of *Galeandra* species. It is dependent on an atomic dating approach. The colonization of the epiphytic specialty of Neotropical woodland canopies played a critical part in orchid’s exceptional broadening, with uncommon reversions to the earthbound propensity. The earthly clade started the synchronously with the rise of dry vegetation biomes within the final 5 million a long time. It is proposing that the aridification is drastically affected the plant diversification and propensities inside the Neotropics. Shift in propensity is affected by the flower lengths and geographic extend the estimate, but not climatic specialty. The longer goads and smaller varieties are needed to characterize the epiphytic species, which are likely adjusted to focusing on extensive tongued Euglossini bees’ pollinators occupying forest propensities (Figure 1) [15].

## Current research trends on the orchid disease

Hsiao et al. [16] have looked into the logical exercises in orchid investigate counting the status of genomics, change innovation, blossoming control and molecular administrative component of botanical advancement, fragrance generation, and color introduction. The endeavors have been made to set up a hereditary change framework in orchids for resistance to orchid diseases. The pepper ferredoxin-like protein quality could be a malady resistance quality, which encodes a ferredoxin-like protein to diminish contamination by *Erwinia carotovora* pathogen for the delicate decay malady [16]. Natural control is another region of malady administration that needs advance to inquire about for orchid plants. *Pseudomonas fluorescens* is an opposing specialist within the soil, viably stifled *F. oxysporum*. A collective vaccination of *Pseudomonas* sp. and *Trichoderma* sp. moreover, is found successful in contrast to *Fusarium* shrink of vanilla [7]. Some study gives modern data concerning about *Dendrobium* orchid pathogens and recognized the five *Fusarium* species recouped from tissue with indications. These discoveries can contribute to way better administration of *Fusarium* infections, which represent a critical challenge to orchid generation in Hawaii [17–19].

A leaf spots illness was far reaching in a plant market of cattleya (*Cattleya lueddemanniana* var. lueddemanniana) in Thailand. The isolated organism caused spot side effects on vaccinated orchid takes off comparative to side effects observed within the field. Typically, the primary description of orchid leaf spots disease is triggered by *N. orchidacearum* [20]. Pathogenicity tests in vitro as well as on tissue-cultured seedlings utilizing parasitic confine GXDF24 appeared that it was able to cause common place dark spot side effects. Molecular distinguishing proof based on the ITS grouping uncovered that confine GXDF24 shared 99% likeness with *Cladosporium oxysporum*. Typically, the primary report of *C. oxysporum* as a pathogen is causing dark spot of *D. officinale* in China [21]. The pathogenicity of the disconnected organism was tried with effective contamination of vaccinated *C. ensifolium* plants. Koch’s hypothesizes were affirmed by reisolation of the organism from the contaminated tissue on misleadingly vaccinated stems. Based on morphological characteristics and arrangement investigation of the inner-deciphered spacer and actin genes, the pathogen was distinguished as *Fusarium oxysporum*. This is often the primary report of *F. oxysporum* causing stem rot in *C. ensifolium* [22].

Fadzil et al. [23] have depicted a picture division strategy for classifying two contrast sorts of orchid leaf illness such as dark leaf spot and sunburn. The orchid leaves pictures were carefully captured by utilizing the advanced camera. With regard to the locale of interest chosen orchid leaves are dissected by utilizing border division strategies utilizing MATLAB (Matrix Laboratory). The graphical client interface has been created to consequently classify orchid infections [23]. Phylogenetic examination based on nucleotide sequences of Coat Proteingene appeared that *Odontoglossum* virus has similitude to *Odontoglossum* ringspot infection Germany, while, other *Odontoglossum* virus lead to speciation that conceivably to be a modern strain. This consider was demonstrated that *Odontoglossum* ringspot infection have crossed and spread broadly by contaminated orchids in nursery, semi-natural timberlands (botanical gardens) and common woodland (national stop) in Java and Bali, Indonesia [24]. More recently, Cating et al. [25] have depicted the pathogenicity specificity of *P. palmivora* on *Dendrobium, Phalaenopsis, Cymbidium,* and *Epidendrum* plants utilizing mycelia of *P. palmivora* on potato dextrose agar (PDA); the inoculum is put on orchid leaf edges and roots. The inoculated plant fabric was put on sterile, sodden blotching paper in polystyrene boxes and hatched at 22 to 25°C within the dark. Small dark injuries were observed at the point of inoculation, and after 3 days, the illness progress causing influenced tissue to seem water-drenched and dark in color [25–28].

## Geographical distribution of orchid disease

**Figure 2.**
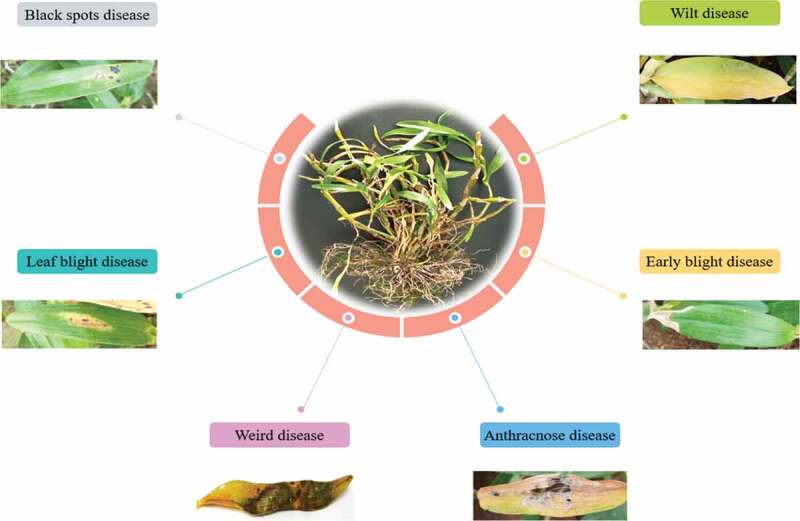
Different type of disease pattern in the Orchids plants

**Figure 3.**
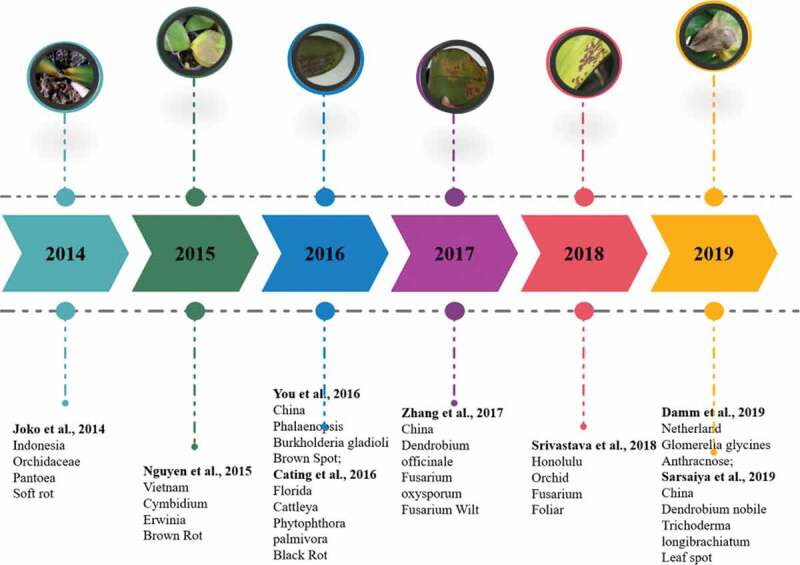
Yearly systematic evaluation of orchids disease with their host and disease name

This section provides a historical overview of the disease and covers several aspects related to hosting range and geographical distribution of the pathogen, current trends for its isolation and detection, and epidemiology and management of the disease as presented in Figures 2, 3 and Table 1. *Fusarium* is one of the major diseases causing pathogens contaminating orchids that are spreading through worldwide exchange. Srivastava et al. [7] have portrayed the *Fusarium* strain as orchids pathogen from Honolulu. Besides the plants, the diseases are moreover, being moved and presented into unused regions. A few *Fusarium* strains related to orchids, a few are pathogenic, which has shown symptoms, for example, leaf and blossom spots, sheath scourges, pseudo-stem or root spoils, and shrinks. Contamination and harm caused by *Fusarium* decreased the quality of plants and blooms, and can cause extreme financial misfortunes. *Fusarium* from orchids are studied worldwide, in spite of the fact that majorities are reported from tropical regions to subtropical parts. Recently, numerous *Fusarium* sp. are known as chief pathogens to orchids, triggering diminished plant potency and diminishing the eminence of pruned plants and its flower. In spite of the fact that a few actions have been shaped to address this issue, they are not adequate. Expanded mindfulness between producers and consumers with respect to the pathogen nature, dealings to avert its development, and conventions infection administration are fundamental for to decrease the pathogen by attaining the objective of disease-free generation [7].

**Table 1.** Systematic evaluation of recent orchid diseases with treatment approaches

Year	Region	Host	Causal Agent	Disease	Symptoms	Prevention	Treatment	Ref.
2014	Indonesia	Orchidaceae	*Pantoea*	Soft rot	Leaves seem yellow, liquid soaked and turn out to be black with sunken	Avoid directly above watering, retain leaves dry, upsurge air flow and decrease temperature and humidity	Instantly eliminate diseased material via disinfected devices, bactericides identical to Physan. Disinfect budding area using 10% bleach solution	[33]
2015	Vietnam	*Cymbidium* orchid	*Erwinia*	Brown Rot	Leaves waterlogged, brown spots turn to black	Circular and brown spots over the exterior of leaves, and light Gray masses spores formed on the rotted zone	Choose resistant varieties whenever possible. Remove infected plant parts, improve air circulation, use earth friendly fungicide and thoroughly spray on the plant.	[13]
2016	China	*Phalaenopsisorchid*	*Burkholderia gladioli*	Brown Spot	The leaf spots were brown, surrounded by yellow halos, usually circular, less often irregular, and they dried. The infection spreads rapidly	Prefers warm, moist conditions. Decrease temperature and humidity, avoid overhead sprinkling and upsurge air flow	Directly eliminate infected material and spray the bactericide like Physan	[34]
2016	Florida	*Cattleya* orchids	*Phytophthora palmivora* and *Phytophthora cactorum*	Black Rot	Initial seem small, irregular, watery, brown spots. Emergence of plant leafs fungal diseases via host shift speciation which speedily turn out to be purplish brown or black	High humidity and temperatures favor the disease spread. Applied the preventative fungicide spray, predominantly in hot humid stages	Eliminate infected material with a germ-free tool, and wet with a appropriate fungicide like Banrot or Subdue	[35]
2017	China	*Dendrobium officinale*	*Fusarium oxysporum*	Fusarium Wilt	Diseased leaves turn yellow, shrill, wrinkled or wilted	Maintain appropriate hygiene. Disinfect cutting tools subsequently each usage, preferably using fire sterilization.	Remove infected portion of pseudobulb and rhizome if the purple color is found. Drench germ-free plants in a thiophanate Methyl	[38]
2018	Honolulu	Orchid	*Fusarium*	Foliar	Flower with leaf spots, sheath blights, wilts, pseudo stem or root rots	Apply water directly around plants by soaker tube, slow successively hose, or soaking can.	Apply water by using the sprinkler in the before noon, resultant foliage dehydrates quickly	[7]
2019	China	*Dendrobium nobile*	*Rhizoctonia*	Fungal Root Rot	Leaves and pseudobulbs converted yellow, shriveled, thin and folded and new developments become gradually smaller. The roots typically display a brown rot along with white/brown fungal development	Fresh potting media for plants are not overwatered. Check their roots and at that time repot as needed. Pots need to flushed at least once-a-month to avoid root damage by soaking heavily	Remove infected part of roots and leaves using a sterile cutting tool, drench the residual plant in a effective fungicide corresponding to thiophanate methyl	[3]
2019	Netherland	Orchidaceae	*Glomerella glycines*	Anthracnose	Leaf edge become brown at the top and spread to the base. Shadowy brown or bright gray areas grow, occasionally as concentric circles or as abundant dark possess transversely the leaf.	Regular sanitation, virtuous air measure, lesser temperatures and amplified light.	Spray systemic fungicide like thiophanate methyl or protectant fungicides like Mancozeb. Alternate systemic and protectant fungicide use.	[36]
2019	China	*Dendrobium nobile*	*Trichoderma longibrachiatum*	Leaf spot	The minor spots started on leaf, endure to expand, turn dark brown to black, circular or irregular lesions.	Maintain clean air, decrease humidity, and water	Eliminate infected parts with a germ-free instrument and decrease leaf moisture. Spray with a complete fungicide.	[37]

Komínek et al. [29] have depicted the plants of the sort Pleione, instigating from leisure activity of agronomists within the Netherlands and within the Czech Republic, are obvious for virus-related contamination, appearing indications of leaf mosaic or blossom defiance. Utilizing Sanger high throughput sequencing, the complete genome arrangement of an original poty virus are gotten from modern sequencing information. The genome sequence was explained and related to the genome of further poty viruses. The genus Poty infection, family Potyviridae, are contaminating the class Pleione. Its complete genome grouping has been categorized, conjointly its transmission via aphids has been illustrated [30,31]. Orchids of the sort *Pleione* D. Wear are little epiphytic, lithophytic, or earthbound orchids. They make yearly pseudobulbs through one or else two leaves, ordinarily deciduous in the wintertime. Blossoms seem either sometimes or later the leaves (generally 1–2/plant). The virus-related pathogen is existing in ailing orchids in cooperation of agronomic attention. There are restricted quantity of systematic information on the orchids pathogens from the genus *Pleione*. Only one infection species, specifically *Pleione* virus Y, is studied to contaminate *Pleione formosana* [29].

Ong et al. [32] have depicted the Novel and unique viruses related to Australian orchid-fungus Symbioses from Australia. Leaves and stems are taken from *P. Sanguinea* plant in Western Australia. It is found that the Earthbound orchids are represented as an advantageous union between plants and mycorrhizal parasites. The event and nature of infections related to one populace of wild *Pterostyl* are enthusiastic orchids, counting their parasitic symbionts. In orchid leaf tissues, it is found that three separates of a different toti virus and an uncategorized virus; both look like fungus contaminating viruses. The plant-fungus advantageous association is critical, but the part viruses may play in this relationship stay generally obscure. Most myco viruses show up to have a small impact on parasitic pathogenicity. Australian earthbound orchids vary from other composite life forms such as lichens in that the relationship is broken every year when the plant partner enters its torpid below-ground stage, and it gets to be reestablished when the shoot reemerges, which may happen up to a few a long time afterward, depending on natural conditions. Pterostylis plants continuously set up mycorrhizal connections with species of *Ceratobasidium parasites*. The foremost common viruses are *cymbidium mosaic virus* (CymMV) and *Odontoglossum ringspot virus* (ORSV) from families Alphaflexiviridae and Virgaviridae. Virus contaminating uninhabited orchids are distant less well identified. In India, confines of ORSV (sort Tobamovirus), CymMV (class Potexvirus), and a novel poty virus-contaminated wild epiphytic orchids. In Japan, the uninhabited Calanthe izu-insularis plants were tainted with cucumber mosaic virus [21] (Table 1).

## Signs and symptoms of orchid diseases

Orchids are grown in numerous sizes, colors, and growth forms. The majority of these wonderful plants are cultivated to come from rainforest areas where temperatures are temperate in tropical. There are also species, which thrive in arid conditions, but these are not widely grown. Orchid plant diseases are most likely to occur when excess moisture stays on leaves and flowers, and when soil has poor drainage. Cultural changes and even a site transfer can minimize disease as can good sanitation procedures. Zhang et al. [38] have described the major symptoms of *Fusarium* wilt disease in *Dendrobium officinale* caused by *Fusarium oxysporum*. The wilt disease has developed a yellow leaves, defoliation arises. In due course, the basal stem along with fibrous roots has become brown, leaves wilted before dropping and plant dies. *Dendrobium officinale* is an old Chinese herb that has both ornament worth and a wide-ranging variety of therapeutic properties. The casual organism of this disease is essential to the development of effective and economical management practices. *Fusarium* sp. of orchids have been described from several places around the biosphere.

**Figure 4.**
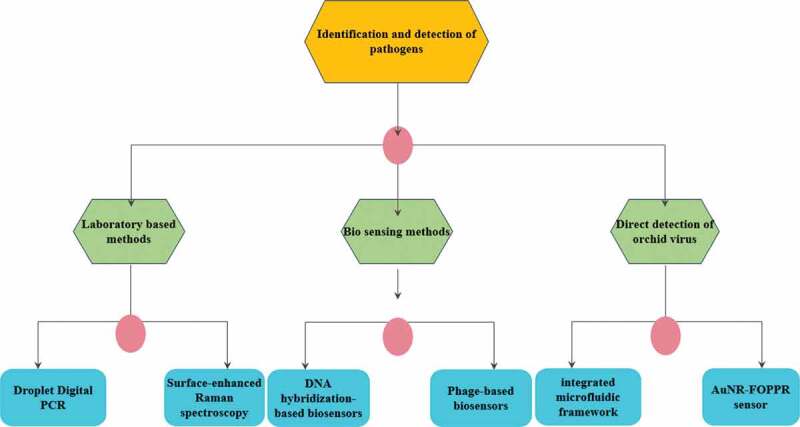
Modern identification approaches for the detection of pathogens

Suwannarach et al. [20] have described the symptoms of leaf spot on cattleya orchid. First appear on the underside as small, water-soaked, irregular spots that are yellowish brown in color, then turn brown or black with a yellowish margin. Under high temperature and humidity, the lesions rapidly enlarge and become soft with ooze if pressed. This disease is caused by *Neoscytalidium orchidacearum*. Leaf spot caused by phytopathogenic fungi are among the key disease affecting Orchidaceae plants worldwide (Figure 4 and Table 2).

**Table 2.** Description of sign of common orchid disease

Common disease signs and symptoms	Key description
Orchid has become a large plant but without flowers or with few barbs.	This is usually a sign of environmental problems. Check lighting, temperature, etc.
The leaves are very dark green.	The dark green leaves are regularly a pined for highlight of an indoor plant, in case an orchid appears dark green leaves it means it isn’t accepting the correct amount of light. Orchid leaves in great wellbeing are olive green.
Brown tips seemed on the orchid leaves.	Disease triggered by excessive fertilization as well as fungal diseases are common causes of leaves darkening.
The orchid leaves seem to be injured by the sun; yellow, calloused, in the mid of the leaves.	Orchids prefer indirect light. Revelation to straight sunlight may cause harm to the leaves.
The orchid leaves have a wrinkled presence.	This is dehydration caused via an irrigation problematic. The plant basically has not been supplied with adequate water or over watered and then rotted roots.
The roots are changed from being white/grayish green to actuality wrinkled	Occurred due to most probable an irrigation problem. If an orchid is not receipting the appropriate volume of water, the roots may initiate to wrinkle.

Xiao et al. [21] have depicted the indications of dark spot on *Dendrobium officinale* triggered by *Cladosporium oxysporum*. The disease shows itself as little, dark spot on leaves, which can develop into circular streaks that gotten to be necrotic tan or brownish dark, with the leaves inevitably turning yellow or sometime brown yellow. Dark spot is common disease stirring on *Dendrobium officinale*, which altogether decreases the value and profit of this medicinal plant. Jin-Ai et al. [22] have depicted the indications of stem spoil on *Cymbidium ensifolium* triggered by *F. oxysporum*. At first, the intersection of the stem and root turns dark black, and the foot leaves turned yellow. Hence, the leaf base started to decay, and the stem turned brown and the whole stem gotten to be necrotic. *Cymbidium ensifolium* is an imperative-developed herb in southern China.

Sudarsono et al. [39] have depicted the soft rot disease may be a destroying disease infecting *Dendrobium phalaenopsis* and *Phalaenopsis* sp. caused by *Erwinia chryysanthemi*. Ordinary soft rot indications showed up regularly on young plants of *D. phalaenopsis* and *Phalaenopsis* sp. soft rot indications as a rule appeared on old leaves of *D. phalaenopsis*, and expanded into entirety leaves, going with blighting of entirety plant. Indication started as a little water-soaked injury on old takes off of *Phalaenopsis* sp., which extended quickly on the clears out and inevitably in soft rot of entirety plant. In warm temperature and high humidity, soft rot disease recurrence is rapidly spread within the field.

## Modern process advancement for the identification and detection of pathogens

### Laboratory research trends

#### Droplet polymerase chain response (PCR)

Droplet PCR (dPCR), could be a later innovation that has ended up commercially accessible since 20,119. The dPCR innovation utilizes Taq polymerase in a standard PCR response to open up a target DNA part from a complex test utilizing pre-validated preliminary or primer/probe tests. It is a supreme evaluation framework that disposes of the require for ordinary bends stabilization. dPCR is specifically compared for quality expression investigation utilizing low amounts of decontaminated, engineered DNA in well-characterized tests beneath indistinguishable response conditions. The strategy requires a costly thermo cycler and components which confines it utilized for on-site pathogens location. The bases of strategies are the exponential enhancement of DNA, permitting to produce lots of particles from a solo molecule, in fair a number of minutes. The polymerase chain response (PCR) is the foremost well known in vitro nucleic-acid amplification approach [40].

#### Surface-enhanced Raman spectroscopy

Surface-enhanced Raman spectroscopy (SERS) is a nondestructive, developing laser-based expository procedure based on Raman scrambling that has been appeared to be a valuable apparatus for the quick location and evaluation of biotic and tests with surface-enhanced Raman spectroscopy. SERS has been broadly connected utilizing resistant tests and atomic tests for pathogen checking in people, plants, creatures, nourishment, water, and the milieu due to its quickness, affectability, and strength for location of biomarkers. Biosensors based on transitory detecting have risen as an extraordinary choice for the convenient point-of-care conclusion much appreciated to their capability, among others, of miniaturization, multiplexing, label-free discovery and integration in lab-on-chip platforms [41].

### Bio sensing approach

Within the arena of diagnostics and biosensing approaches are supplanting conventional discovery strategies as a result of their little measure of unit and compactness. The nanowire-based biosensor application may be the most dependable sensor these days since of focal points toward recognizing natural atom particularly plant illnesses. Recently, analysts are utilizing an coordinates approach by combining nanosciences innovation, hardware, computers, and science to form versatile biosensors with exceptional detecting capabilities that appear uncommon spatial and transient determination and unwavering quality.

#### DNA hybridization-based biosensors

DNA hybridization biosensors have also been detailed that given PCR-free visual nucleic corrosive location offering effortlessness, high affectability, and fast results. Recently, a nano propelled biosensor, within the arrangement of DNA-based diagnostics, the localized surface plasmon reverberation of gold nanoparticles is detailed to be utilized to create a colorimetric nano-biosensing framework to distinguish the unamplified genome in contaminated plants. RNA (ribonucleic acid) targets can moreover, be recognized utilizing turn around the transcriptase (RTase) protein which changes over the RNA into cDNA (complementary DNA) earlier to hybridization. Afterward, the transducers change the detected DNA target into the signals which are advanced expand by an enhancer. When preparing a DNA biosensor, the aptamer generation, which needs single-stranded and 15–40 bases long DNA or RNA oligonucleotide groupings, is of imperative significance. The aptamer generation is based on orderly advancement of ligands by exponential improvement and polymerase chain response (PCR). DNA-based electrochemical biosensors depending on a redox response, exchange electrons among cathodes after hybridizing the target to an immobilized detention test, though the electrochemical label permitted biosensors rely on the alter resistivity. The improvement of beneficial biosensing frameworks for plants pathogen discovery are depending on both counteracting agent and DNA receptors. The utilization of distinctive nanomaterials for example nanochannels and metallic nanoparticles for the advancement of imaginative and delicate biosensing frameworks for the discovery of pathogens (microbes and infections) is additionally appeared [42].

#### Phage-based biosensors

Phages are utilized to manage bacterial plant contaminations; though, as a result of their authoritative partiality to bacterial compartments, carbohydrates, or proteins, phage are utilized to analyze contamination. The interface among phage and the focused bacterial constituent carries the response which is changed over the quantifiable signals via the transducer. The phage-based biosensors are exceptionally delicate, more effective, quick, and strong and they show a extended lifespan due to their steadiness at a higher temperature. These biosensors are competent in identifying practical pathogens. For plant pathogen discovery, phage-based magnetoelastic (ME) biosensors are technologically advanced for the discovery of *S. Typhimurium*. These phage-based biosensors have demonstrated to be more sensitive and steadier [43].

### Direct detection of orchid virus

Chang et al. [44] depicted the integrated microfluidic framework could be a promising stage to distinguish Phalaenops is orchid infections straightforwardly from new leaves. This was illustrated utilizing four common plant infections. With this microfluidic framework, unpractised technicians can perform the pathogen discovery trends within the field since it is a programmed framework and the device does not require a bulky gel electrophoresis or fluorescence location unit. This microfluidic loop-mediated isothermal amplification (LAMP) system encompasses a location restrain of 35 pg. Usually the primary time an coordinates microfluidic framework was detailed to, straightforwardly degree from new takes off, pathogens common to the Phalaenopsis orchid. In-field agrarian checking may significantly advantage from this promising apparatus within the near future.

Lin et al. [45] have illustrated the possibility of gold nanorods-fiber optic particle plasmon resonance sensor for refractive index detecting and label-free orchid infection discovery in weakened plant unrefined saps. The potential applications of the gold nanorods-fiber optic particle plasmon resonance sensor are not constrained to the discovery of a single orchid infection, but may be expanded to multiplex discovery by fitting the angle proportion of gold nanorods-fiber optic particle plasmon resonance to create a sum of spectral detecting windows from the noticeable to the near infrared section. Wylie et al. [46] have depicted the ought to isolate recently collected propagules until plants are examined for indications, and in a perfect world measured for infections some time recently presenting them to ex situ preservation or green collections. In captive populaces where plants are developed in pots on seats, conditions exist for the fast spread of infections by vectors. The case of TuYV is of specific concern since the infection is likely extraordinary and obvious indications of disease were not clear. Disposal of outlandish infections from undermined orchid populations is certainly covetous for their long-term survival. But within the plausible cause of the partitivirus, it is questionable whether any of the recognized infections are transmitted through seed and pick up a more prominent understanding of the role’s infections play in long-term organizations with plants, especially for plants of preservation concern. Critically, preservation programs for orchids have considered infection disease, but ordinarily, as it were related, with epiphytic taxa. This investigated the biological parts of inborn and outlandish infections as well as evaluate phyto-health dangers to preservation of ex situ and wild orchid populaces.

Kondo et al. [47] have depicted the Orchid fleck virus (OFV), which features a two-segmented negative-strand RNA genome and takes after plant nucleorhabdo viruses, remains unexplored. The transcripts of six genes encoded by OFV RNA1 and RNA2 within the poly (A)-enriched RNA division from infected plants were molecularly characterized. All of the OFV mRNAs were started at a begin arrangement with one to three non-viral adenine nucleotides which were included at the five closes of each mRNA. The nearness of polyadenylated brief transcripts inferred from the three terminal pioneer locales of both genomic and antigenomic strands, giving the primary example of also- and minus-strand pioneer RNAs in a fragmented minus-strand RNA virus. The similitude within the transcriptional technique between this bipartite OFV and monopartite rhabdo viruses, particularly nucleorhabdo infections (family Rhabdoviridae) is extra back for their near relationship.

## Modern biocontrol and prevention measurements

Buying disease-free orchids from a legitimate nursery and giving those orchids with their perfect social conditions is the finest avoidance strategy. Buy orchids developed from seed at whatever point conceivable since these are known to be disease-free. Separate unused orchids until to know these are disease-free. Sterilize the devices when it is pruning, proliferating, or collecting orchid blooms. The most perfect way to sterilize metal tools is to plunge them in 70% rubbing liquor and after that utilize a fire to encourage sterilize them. The least demanding strategy of avoiding the spread of leaf infection in orchids is to annihilate the influenced plants, preferably by burning them. Never include the infected plants for compost heap. Be careful for virus-carrying insects and shower or disinfect as required to slaughter them. Provide orchids with perfect social conditions and maintain a strategic distance from common mistakes like over or beneath watering. Employing a clean and sterile apparatus or edge, cut the contaminated leaf back until exposing the sound tissue. Treat the great edge of the leaf with a bactericidal arrangement. Be sure to put the orchid plant in a zone where it can reach great air circulation; usually to guarantee that the bactericidal leaf edge dries appropriately. It is required to create sure arrangement for dries rapidly and makes a seal over the injured region. Take after these tips for control the infection:

- Relocate such orchid uncover it to superior air circulation, lower humidity, and temperature between 65 and 80 degrees.
- Spray such orchid with a great quality, broad-spectrum fungicide, taking after bundle headings. in the event that its bacterial infection, fungicide application will avoid auxiliary infection.
- Eliminate the disintegrated or unhealthy parts of the plant at all times by trimming them and arrange them afterward.
- Heal the wounds with coal dust, which could be a characteristic disinfectant.
- Wash hands completely before and after continuing to kill any infected zone, so as not to transmit illnesses from one plant to another.
- If it is reutilized the pots, this can be fine, but need to clean them altogether with hot soapy water.
- Before continuing to dispense with any portion of the plant, it must purify the cutting apparatuses well, such as scissors and knives.

### Nanotechnology used for controlling plant diseases

Nanotechnology is one of the foremost interesting and quickly progressing sciences and has potential to revolutionize numerous disciplines of science, innovation, pharmaceutical, and horticulture. Nanoparticles can be delivered by diverse strategies, chemical and natural, the previous is commercially utilized. Nanomaterials can be possibly utilized within the edit security, particularly within the plant infection administration. Nanoparticles may act upon pathogens in a way comparable to chemical pesticides or the nanomaterials can be utilized as carrier of dynamic fixings of pesticides, have resistance actuating chemicals, etc. to the target pathogens. Since of ultra-small measure, nanoparticles may hit/target infection particles and may open a unused field of infection control in plants [48]. Nanoparticles are too successful against bothers or creepy crawlies. Silica nanoparticles have been utilized as smaller-scale supplements (included in plant development and direction of push) as well as being viable against bacterial contaminations. The association of nanoparticles with botanical compounds can back bug administration by giving insecticidal and repellent movement. Nanoparticles can have significant impacts on rhizosphere work, causing changes within the generation of key metabolites that contribute to plant security against *Pseudomonas chlororaphis* [49].

#### Nanosilver

Nanosilver is the foremost considered and utilized nano molecule for bio-system. It has long been known to have solid inhibitory and bactericidal impacts as well as a wide range of antimicrobial exercises. Silver nanoparticles, which have tall surface region and tall division of surface molecules, have high antimicrobial impact as compared to the bulk silver. Antifungal adequacy of colloidal nano silver (1.5 nm normal distance across) arrangement against fine mold caused by *Sphaerotheca pannosa* Var rosae. It could be an exceptionally wide spread and common malady of both green house and open-air developed plants. It causes leaf twisting, leaf twisting, early defoliation and decreased blooming. Twofold capsulized nanosilver was arranged by chemical response of silver particle with help of physical strategy, lessening specialist, and stabilizers. They were exceedingly steady and exceptionally well dispersive in watery arrangement. It disposes of undesirable microorganisms in grower soils and hydroponics frameworks. It is being utilized as foliar splash to halt parasites, molds, decay, and a few other plant diseases. Additionally, silver is an amazing plant-growth stimulator. nanosilver as nano pesticides gives a comprehensive see on utilize of nano silver for battling plant infections [50].

#### Nanocopper

The nanolevel copper is shown a antimicrobial (bacterial and fungal) properties. Consequently, the utilize of nano copper is additionally predominant to regulate plant diseases. The synthesized copper composites are used as exceptionally compelling antibacterial operators and were able of altogether diminishing bacterial spot disease in plants. Nanocopper exposed plants are appeared predominant as soil macronutrients. The characteristics of organisms within the union of nanoparticles for uses in plant infection controller. These nanoparticles were moreover exceptionally compelling when silver and copper nanoparticles are mutually organized. Nanoparticles had inconvenient impact on fungal hyphae development and conidial germination as uncovered by the infinitesimal perception. nanoparticles are assessed in contrast to two pathogenic organisms *Alternaria substitute* and *Botrytis cinerea*, which are capable of triggering harm to expansive numeral plants and used copper nanoparticles for the control of both pathogens [51].

#### Metal oxide nano particles

Metal oxide nanoparticles are recognized as different types of nanomaterials. These are widely used for the treatment of plant pathogens such as antifungal action of zinc oxide (ZnO) and magnesium oxide (MgO) nanoparticles on *A. interchange, F. oxysporum, R. stolonifer* and *M. plumbeus*. The adequacy of these nanoparticles was too tried for actuating complete resistance and protection in contrast to bacterial wilt disease. The interaction of MgO nanoparticles with bacteria, hence harming the bacterial exterior, are used to examine the antibacterial action of such nanoparticles. Nano-inorganic metal oxide encompasses a possibility to decrease bacterial defilement. MgO is an imperative inorganic oxide and are broadly utilized in numerous areas [9]. Sulfur nanoparticles, silica nanoparticles, carbon-based materials and polymer composites have also shown the powerful antimicrobial and antifungal properties and subsequently can be viably utilized for governing plant disease. In this respect, Rao et al. [52] stated fungicidal possessions of sulfur nanoparticles against two phytopathogens, *F. solani* accountable for causing initial blight disease and *Fusarium* wilt disease [53].

## Challenges, needs, and future outlooks

Many genera and inter generic orchids hybrids are cultured for the production of flowers, sprays, and natural compounds. The chief challenge for these studies is to grow huge orchids populations for evaluation and testing. These crops are both very slow budding and expensive. Diseases of orchids are mainly triggered by bacteria, fungi, and viruses. They are classified as leaf spots; flower blights; and root, stem and pseudobulb rots, which are the most serious. Due to their complex biology, notably their interactions with pathogenic and nonpathogenic microbes, orchids present particular challenges for conservation, and this is compounded by non-sustainable and often illegal collection for horticulture, medicine, and food and by climate change [54]. Maintaining or increasing the yield of orchid variants through classical breeding is challenging because productivity is limited by infection of the pathogenic microbes that cause orchid diseases. To unravel this issue, endeavors have been made to set up a hereditary change framework in orchids for resistance to orchid diseases. Transgenic orchid plants appeared upgraded resistance to pathogenic microbes’ infection. This can be the primary report depicting a transgenic *Phalaenopsis* orchid with double resistance to phytopathogens [16].

*Fusarium* species are commonly reported in affiliation with disease, studies to characterize a pathogenic relationship are uncommon [17]. Fungicide resistance could be a major issue in disease administration programs. Fungicides with exceptionally particular destinations of movement are more inclined to the advancement of fungicide resistance than fungicides which have broader extend of antifungal activities or numerous destinations of action. In this way, testing to distinguish extra fungicides that viably control Fusarium is direly required. The greatest challenge for such studies in any case, is to develop huge populaces of orchids for assessment and testing, as this edit is both exceptionally moderate developing and costly. Recognizable proof of *Fusarium* species is one of the most prominent challenges for pathologist. *Fusarium* scientific classification is continually changing for years and proceeds to alter, so assigning a name to a specific species can be both troublesome and questionable [7].

The key challenges are to evaluate the effect of inadequate or inaccurate host information on the prescient precision of models, and to create strategies to account for the extra vulnerability to which this lead. Misfortune of small-scale spatial detail regularly makes artificially-extended districts in which the host may wrongly be expected to be coterminous; the potential predisposition of this in exaggerating spread remains unclear [55]. Morphological recognizable proof of OFEs (Orchidaceae fungal endophytes) to species or once in a while indeed class position isn’t persistently plausible. Numerous OFEs are not sporulated, indeed when sporulation empowering strategies are utilitarian. There are a few drawbacks in relying on bioinformatics strategies for classifying endophytes, counting low esteem, and misidentification of ITS groupings by GenBank since ITS arrangements in GenBank are wrongly named [3]. The prevention and treatment of disease resistance may be an extraordinary challenge upgraded by the complexity of this pathogenesis. Angiogenesis plays a major part in the pathogenesis of proliferative malady resistance [56].

Targeted microbiome building for crops could be a future trend. Biodiversity should be a biomarker for this microbiome tweaks. Higher plant-associated differing qualities can be accomplished not as it were through the execution of natural control operators which shifts the microbiome, but too by the application of microbial consortia. In this context, crop-specific natural consortia can be taken together from a pool of chosen biocontrol specialists [57]. In expansion, pathogens such as viruses, viroids, fungi, and phytoplasma truly decrease the generation of chrysanthemums. Transcriptome-sequencing innovation has moreover been utilized to look at disease resistance to chrysanthemum black spot disease, a few viral diseases, and white-rust disease [58]. Such studies have emphasized that co-inoculation of two or more pathogens reliably cause more inconvenient impacts on root advancement than either pathogen alone. These discoveries will direct future inquire about damping-off diseases, counting studies of the hereditary differences inside species, epidemiological and biological highlights of the disease, and host–pathogen interactions, and eventually help to develop durable and economical damping-off administration practices [59]. OFEs have numerous biotechnological future conceivable outcomes as normal metabolite makers should be measured when planning upcoming applications to spare endangered Orchidaceae. OFEs may be found as a treasure metabolites over possibly exploitation of biotechnological methods attentive on the imperiled form of Orchidaceae within the adjacent forthcoming [3].

## Conclusion

Orchids have continuously been a crop of financial significance. With expanding around the world request for these excellent and outlandish plants, numerous nations have started developing and sending out orchids. Expanded generation and worldwide exchange have driven to the development of Plants. Nanotechnology in this way offers exceptionally compelling strategies to regulate plant diseases. Basic investigation of the investigation done so distant within the field of utilization of nanoparticle for monitoring plant diseases evidently recommend that nanoparticles like nano silver, nano copper, metal oxides, and nano definitions appear exceptionally powerful antibacterial and antifungal properties. These nanomaterials are moreover exceptionally viable at lower concentration and distant way better choices than routine pesticides. There are a few types of rot diseases simply should look out for when caring for an orchid plant. A few of the more common diseases influence not only the leaves, but the buds and roots as well. In the event that cleared out untreated plant might be confronting death, or more regrettable, it seems to spread to other plants in domestic or office (resulting within the death of several plants, instead of just the plant that it originated from). The most excellent care is always prevention, so make beyond any doubt that simply are tending to orchid plant routinely, and giving it the ideal care, it must develop and remain solid. Studied on and learn almost this particular type of rot, and how to spot, and treat it. In spite of the fact that a few measures have been developed to address this problem, they are not adequate. Expanded awareness among producers and buyers with respect to the nature of this pathogen, measures to anticipate its development, and convention disease administration are basic for to decrease the spread of the pathogen and to realize the objective of disease-free orchid generation.

## Research Highlights

- To critically analyzed the global distribution of Orchidaceae diversity and its disease patterns;
- To evaluate the identification and detection of the microbial disease process;
- Important study for the development of best operative disease management plan.
